# Multi-omic based molecular profiling of advanced cancer identifies treatable targets and improves survival in individual patients

**DOI:** 10.18632/oncotarget.26198

**Published:** 2018-10-05

**Authors:** Alexandra Samsen, Silvia von der Heyde, Carsten Bokemeyer, Kerstin A. David, Bernd Flath, Max Graap, Bianca Grebenstein, Ludger Heflik, Wiebke Hollburg, Peter Layer, Eike von Leitner, Friedrich Overkamp, Wolfgang Saeger, Sandra Schneider, Cay-Uwe von Seydewitz, Axel Stang, Alexander Stein, Carsten Zornig, Hartmut Juhl

**Affiliations:** ^1^ IndivuTest GmbH, Hamburg, Germany; ^2^ Indivumed GmbH, Hamburg, Germany; ^3^ II. Medical Clinic and Polyclinic, Department of Oncology, Hematology, Bone Marrow Transplantation and Pneumology, University Cancer Center Hamburg, Hubertus Wald Tumorzentrum, University Medical Center Hamburg-Eppendorf, Hamburg, Germany; ^4^ HOPA-Hämatologisch−Onkologische Praxis Altona, Hamburg, Germany; ^5^ Praxis Und Tagesklinik Für Internistische Onkologie und Hämatologie, Recklinghausen, Germany; ^6^ Israelitisches Krankenhaus, Hamburg, Germany; ^7^ Oncologianova GmbH, Recklinghausen, Germany; ^8^ Medizinische Klinik, Onkologie/Hämatologie Und Palliativmedizin, Krankenhaus Reinbek St Adolf-Stift, Reinbek, Germany; ^9^ Department of Hematology, Oncology and Palliative Medicine, Asklepios Klinik Barmbek, Hamburg, Germany

**Keywords:** molecular profiling, advanced stage IV cancer, MAPK pathway, PI3K/AKT/mTOR pathway, targeted therapy

## Abstract

A proof-of-concept study was conducted to assess whether patients with advanced stage IV cancer for whom predominantly no standard therapy was available could benefit from comprehensive molecular profiling of their tumor tissue to provide targeted therapy. Tumor samples of 83 patients were collected under highly standardized conditions and analyzed using immunohistochemistry, next-generation sequencing and phosphoprotein profiling. Expression and phosphorylation of key oncogenic pathways were measured to identify targets at the (phospho-) proteomic level. At genomic level, 50 oncogenes and tumor suppressor genes were analyzed. Based on molecular profiling, targeted therapies were decided by the attending oncologist. Accordingly, 28 patients who met the defined criteria fell in two equal-sized groups. One group received targeted therapies while the other did not. Following six months of treatment, disease control was achieved by 49% of patients receiving targeted therapy (complete remission, 14%; partial remission, 21%; stable disease, 14%; disease progression, 36%; death, 14%) and 21% of patients receiving non-targeted therapy (stable disease, 21%; disease progression, 64%; death, 14%). Individual patients experienced dramatic responses to a therapy which otherwise would not have been applied. This approach clarifies the value of multi-omic molecular profiling for cancer diagnostics.

## INTRODUCTION

Given estimates that over 800 cancer treatments are currently in clinical development [1], molecular profiling of tumors will likely become increasingly important to enable appropriate treatment decisions to be made. Molecular alterations of genes and proteins in small tumor samples can be identified using modern technologies including next-generation sequencing (NGS), identification of phosphorylated isoforms of signaling proteins using nanofluidic proteomic assays [2, 3] and immunohistochemistry to detect targetable proteins such as growth factor receptors. The use of molecular profiling to understand tumor characteristics is currently the basis for therapeutic decision-making in advanced non-small cell lung carcinoma (NSCLC) [4] and metastatic colorectal cancer [5–7]. However, while most current approaches are based on analysis of either activating mutations or receptor protein expression, the phosphorylation status of signaling proteins is generally not measured and approaches integrating all of these data types are usually not applied. A more holistic view may be achieved by considering the genomic as well as the (phospho-) proteomic level. Such comprehensive molecular analysis and subsequent matching to related therapies has previously been associated with improved outcomes for some patients with metastatic cancer [8, 9]. However, such diagnostic approaches depend on standardized tissue collection with short ischemia time (<10 minutes) because otherwise (phospho-) protein level could artificially change significantly [10].

**Figure 1:**
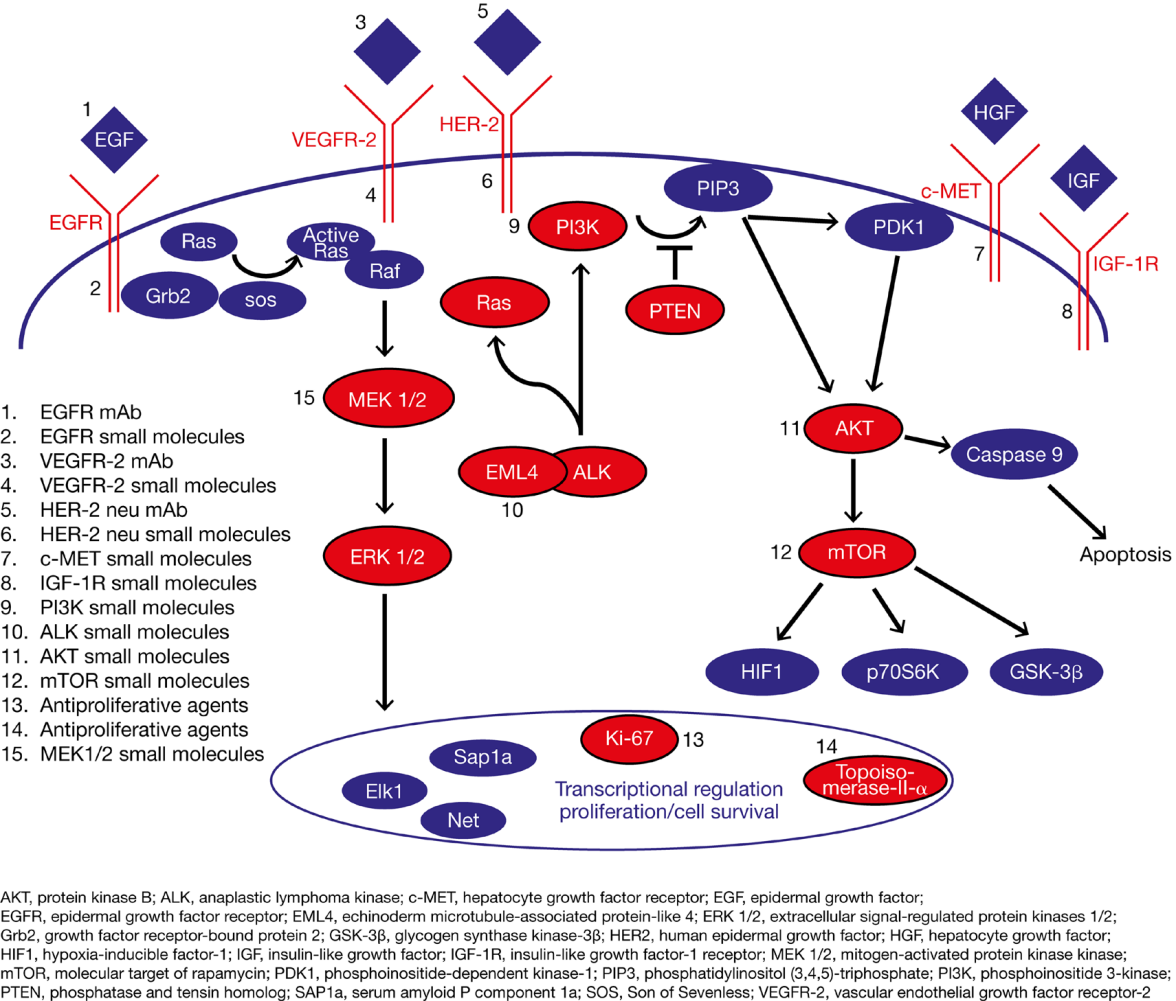
Key oncogenic pathways Overview of relevant pathways and molecular targets investigated in this study; druggable receptor and signaling proteins analyzed by molecular profiling are shown in red.

The aim of this proof-of-concept study was to evaluate whether patients with advanced stage IV cancer, independent of its origin, could benefit from comprehensive molecular profiling of their tumor tissue to enable targeted therapy. Potential drug targets resulting from molecular profiling were summarized in a scientific report for the attending physicians/oncologists who were responsible for therapy selection. The key oncogenes and major signaling pathways in oncogenesis and tumor progression relate to the mitogen-activated protein kinase (MAPK) and phosphoinositide 3-kinase (PI3K)/protein kinase B (Akt)/molecular target of rapamycin (mTOR) signaling pathways [11]. In this study, targeted therapy as determined by molecular profiling was specifically related to “druggable” receptor and signaling proteins associated with the MAPK and PI3K/Akt/mTOR signaling pathways (Figure 1).

## RESULTS

**Figure 2:**
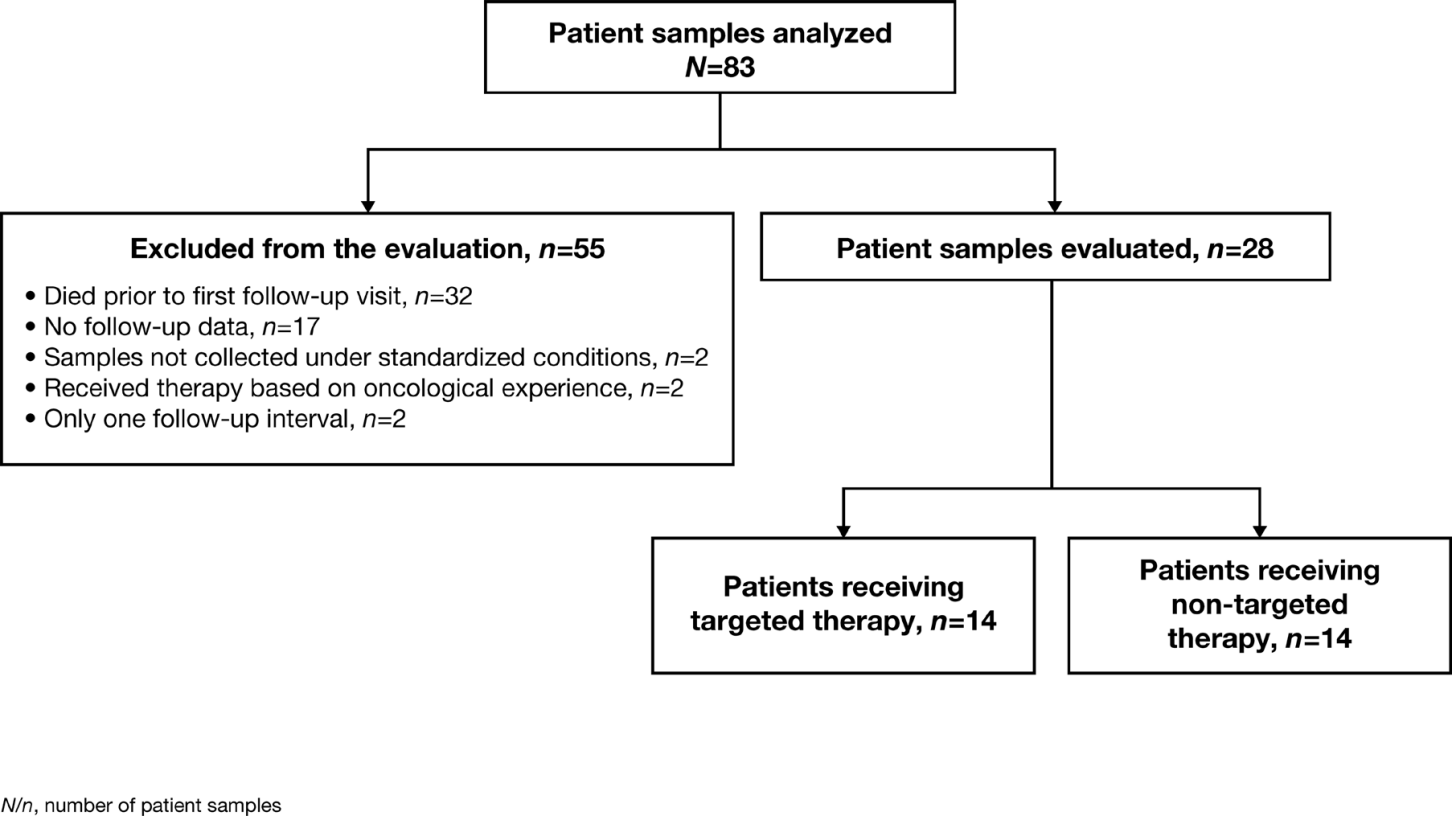
Overview of all 83 patients whose tissue samples were analyzed and the applied selection criteria The 28 patients who passed the filtering criteria and were finally evaluated in detail were divided into two groups according to their initial treatment.

Tumor samples from 83 patients (age range: 25-78 years; mean age 56.6 ± 13.6 years; 36% female) with different primary entities were collected and analyzed using three different methods whenever possible, which was feasible for 51/83 (61%) of patients. Immunohistochemistry, phosphoprotein profiling and NGS was applicable for 82/83 (99%), 52/83 (63%), 73/83 (88%) of patients, respectively. At least one “druggable” target was found for the majority of the 83 analyzed patients with at least one of the described methods, specifically 75/82 (91%) using immunohistochemistry, 18/52 (35%) using phosphoprotein profiling and 21/73 (29%) by applying NGS. To investigate whether the attending oncologist took the identified potential drug target(s) into account for therapy decision and to evaluate the treatment process, follow-up data were collected three months after molecular profiling. Follow-up data were available for 66/83 (80%) patients; however, 38/66 patients (58%) were excluded from the evaluation, due to the fact that the majority of these patients (32/38 [84%]) died prior to the first follow-up interval, two tumor samples were not collected under high-quality standardized conditions, two patients received therapy based on oncological experience and not due to the outcome of molecular profiling, and follow-up data had been collected at only one interval for two patients (Figure 2).

**Figure 3:**
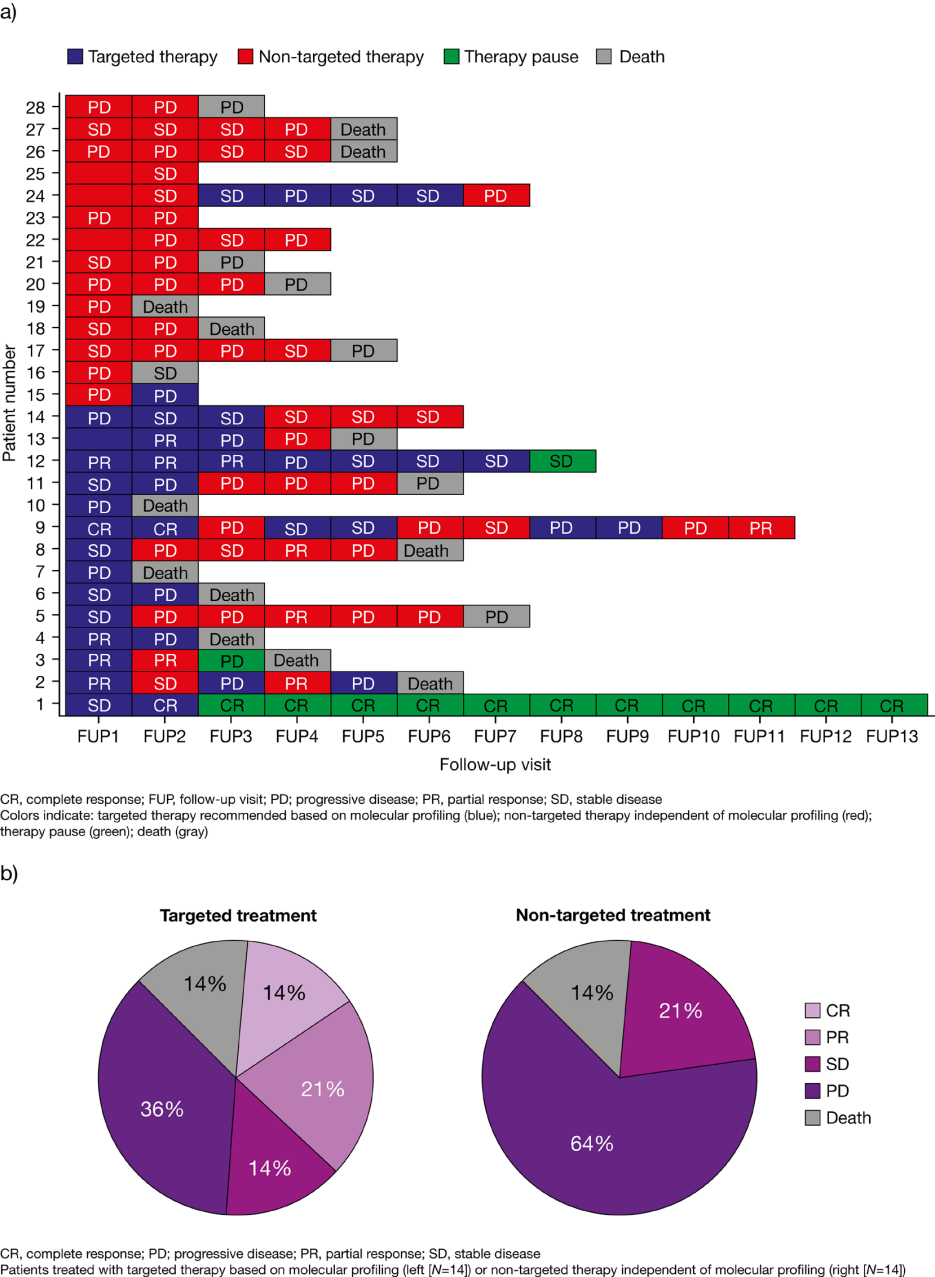
Disease outcome relating to treatment at each follow-up visit **(a)** and tumor response after six months **(b)** in all evaluated patients (*N*=28). Diagram of all 28 evaluated patients in relation to their follow-up frequency. The first follow-up (FUP1) was raised three months after report generation and was requested in three months intervals. Blue bars: Recommended drugs were considered during follow-up interval. Red bars: Patient was treated with other drugs than recommended. Green bars: Therapy was interrupted. Grey bars: Patient died. Response to treatment in each interval is described by abbreviations: Stable disease/Minor response (SD), Partial Response (PR), Complete Response (CR) and Progressive Disease (PD) (a). Pie chart of treatment response after six months in the second follow-up interval (FUP2). The left panel shows the response of 14 patients to treatment following the targeted treatment recommendation, while the right panel shows the response of the remaining 14 patients to other treatments. The following abbreviations were used: Progressive Disease (PD), Stable Disease/Minor Response (SD), Partial Response (PR), Complete Response (CR) (b).

The remaining 28 patients were divided into two groups according to the initial follow-up data (general overview of data in Figure 3a and 3b). The distribution of the tumor types of the evaluable patients was: colorectal cancer (25%), CUP (14%), single rare tumor types (14%), gastric cancer (11%), cholangiocarcinoma (7%), pancreatic cancer (7%), TNBC (7%), breast cancer (4%), esophageal carcinoma (4%), NSCLC (4%) and renal cell carcinoma (4%) (Table 1).

**Table 1:** Patient demographics, tumor entity and tissue analyzed for molecular profiling

Patient number	Birth Year	Gender	Tumor entity	Analyzed tissue
**1**	1966	M	Gastric cancer	Primary tumor
**2**	1940	M	CCC	Liver metastasis
**3**	1939	F	CUP	Lymph node metastasis
**4**	1968	F	TNBC	Cutaneous metastasis
**5**	1977	M	Anal cancer	Liver metastasis
**6**	1951	M	Gastric cancer	Primary tumor
**7**	1946	M	Esophageal carcinoma	Primary tumor
**8**	1959	M	Rectal carcinoma	Brain metastasis
**9**	1963	F	Breast cancer	Cutaneous metastasis
**10**	1956	F	Cecum carcinoma	Liver metastasis
**11**	1950	F	Tube carcinoma	Liver metastasis
**12**	1967	M	Rectal carcinoma	Lung metastasis
**13**	1965	M	Neuroendocrine pancreatic carcinoma	Liver metastasis
**14**	1992	M	SETTLE tumor	Liver metastasis
**15**	1949	M	CUP	Lymph node metastasis
**16**	1946	M	CUP	Peritoneal carcinosis
**17**	1970	F	TNBC	Cutaneous metastasis
**18**	1962	M	Gastric cancer	Primary tumor
**19**	1976	M	Rectal carcinoma	Liver metastasis
**20**	1941	F	CCC	Liver metastasis
**21**	1948	M	Colon carcinoma	Primary tumor
**22**	1961	M	Acinic cell carcinoma of parotid gland	Chest wall metastasis
**23**	1957	M	RCC	Liver metastasis
**24**	1951	F	NSCLC	Bone metastasis
**25**	1988	F	Desmoid (abdominal wall)	Primary tumor
**26**	1964	F	CUP	Ovary
**27**	1960	M	Pancreatic cancer	Liver metastasis
**28**	1972	M	Colon carcinoma	Liver metastasis

CCC, cholangiocarcinoma; CUP, cancer of unknown primary; F, female; M, male; NSCLC, non-small cell lung carcinoma; RCC, renal cell carcinoma; SETTLE, spindle epithelial tumor with thymus-like differentiation; TNBC, triple-negative breast cancer.

Based on the oncologists' treatment decision, one group (*N*=14) was initially treated with targeted therapy based on molecular profiling while the other group (*N*=14) was treated with non-targeted therapy independent of molecular profiling. The targeted treatment group also contains patients who received chemotherapies. These therapy decisions were also based on the results of the molecular profiling approach, because Ki-67 and Topoisomerase were part of the test and these agents were topoisomerase inhibitors.

**Figure 4:**
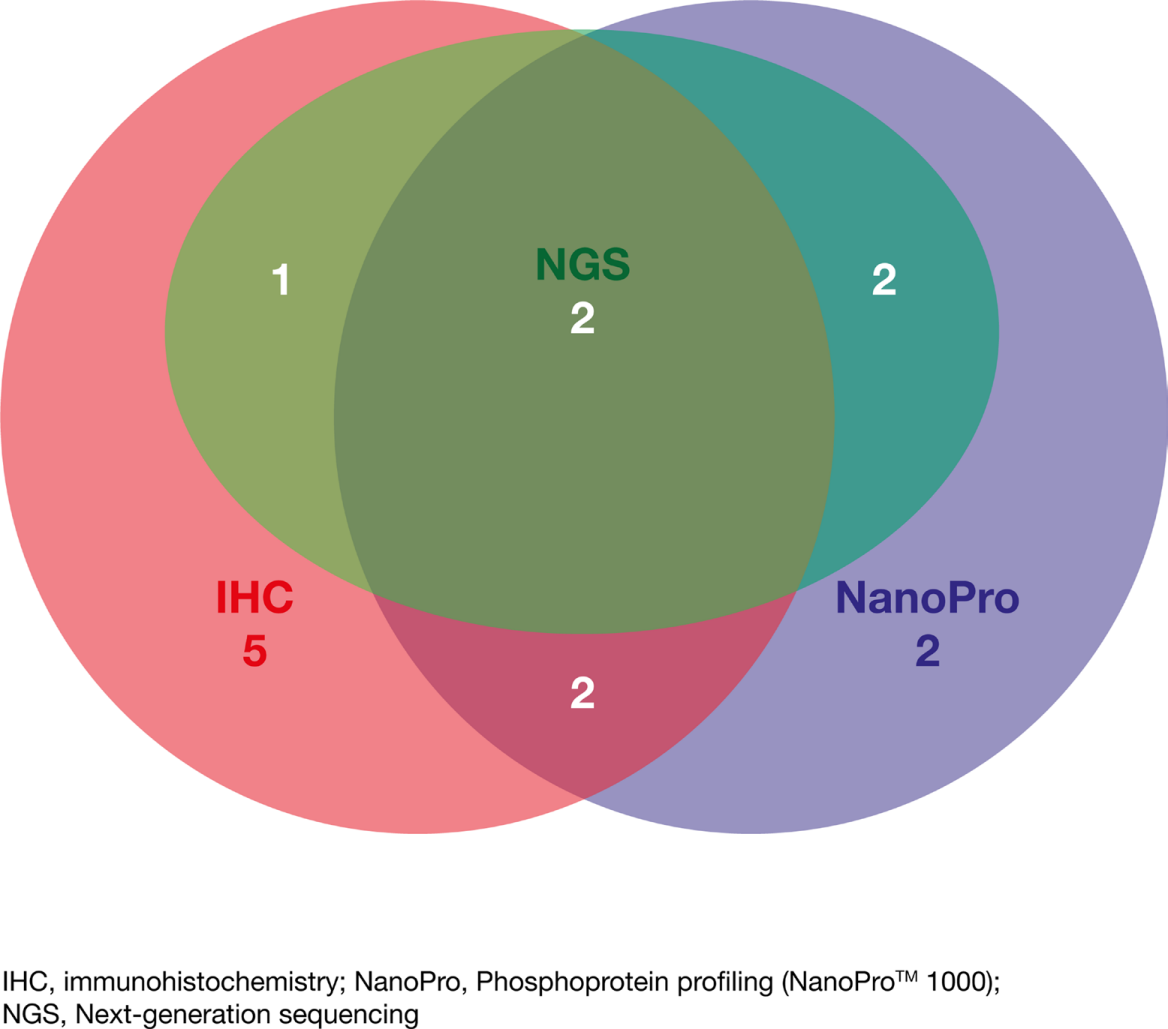
Venn diagram indicating the number of detected targets by each of the different approaches (IHC, NGS and NanoPro) and upon which therapeutic decisions were based for the subgroup of patients treated based on molecular profiling (*N*=14)

With regard to the subgroup of patients who initially received therapy based on molecular profiling, extracellular proteomic alterations detected by immunohistochemistry were identified in 10/14 (71%) patients, intracellular proteomic alterations in signaling pathways were found in 8/14 (57%) patients using phosphoprotein profiling and genomic alterations were identified in 5/14 (36%) patients by NGS. Figure 4 shows the number of detected targets on which decisions for initial targeted therapy were based using the three different molecular profiling methods in the subgroup of patients who initially received therapy based on molecular profiling. A detailed overview of the identified potential drug targets/drug exclusion targets (alterations of proteins and genes) and the initially applied agents directly following molecular testing is summarized for every case of the 28 evaluated patients in Table 2.

**Table 2:** Overview of identified alterations of proteins and genes and applied agents for 28 evaluated patients

Patient number	Identified potential drug targets/drug exclusion targets	Initially applied agents
**1**	Strong HER-2 overexpression, Ki-67 high, strong Topoisomerase-II-α overexpression	Pertuzumab, Trastuzumab and chemotherapy
**2**	Strong EGFR overexpression, RAS wild-type, increased phosphorylation of Akt, activating PIK3CA mutation, activating KIT mutation	Gemcitabine and Cisplatin plus Cetuximab
**3**	Strong EGFR overexpression, KRAS mutation, increased phosphorylation of ERK1/2, MEK1/2 and Akt, moderately strong HER-2 overexpression (2+), no HER-2 amplification	Everolimus
**4**	Increased phosphorylation of ERK1/2, MEK1/2 and Akt, Ki-67 high, strong Topoisomerase-II-α overexpression	Topotecan
**5**	Strong EGFR overexpression, RAS wild-type, increased phosphorylation of MEK1/2 and Akt, Ki-67 high, moderately strong Topoisomerase-II-α overexpression	Panitumumab
**6**	Moderately strong c-MET overexpression, weak expression of EGFR, increased phosphorylation of MEK1/2, Ki-67 high, low Topoisomerase-II-α expression	Folinic acid, Fluorouracil and Irinotecan (FOLFIRI)
**7**	Moderately strong EGFR overexpression, RAS wild-type, increased phosphorylation of ERK1/2, MEK1/2 and Akt, Ki-67 high, moderately strong Topoisomerase-II-α overexpression	Everolimus
**8**	Strong EGFR overexpression, KRAS mutation, Ki-67 high, strong Topoisomerase-II-α overexpression	Regorafenib
**9**	Strong EGFR overexpression, RAS wild-type, strong HER-2 overexpression, activating PIK3CA mutation, Ki-67 high, moderately strong Topoisomerase-II-α overexpression	Trastuzumab and Lapatinib
**10**	Moderately strong EGFR overexpression, KRAS mutation, Ki-67 high, moderately strong Topoisomerase-II-α overexpression	Folinic acid, Fluorouracil and Oxaliplatin (FOLFOX) and Bevacizumab, Regorafenib
**11**	Weak EGFR overexpression, RAS wild-type, Ki-67 high, moderately strong Topoisomerase-II-α overexpression	Irinotecan and Panitumumab
**12**	Moderately strong EGFR overexpression, KRAS mutation, weak VEGFR-2 expression, increased phosphorylation of ERK1/2 and MEK1/2, strong c-MET overexpression, no MET amplification, Ki-67 high, moderately strong Topoisomerase-II-α overexpression	Fluorouracil and Irinotecan and Bevacizumab
**13**	FLT3 mutation, Ki-67 high, low Topoisomerase-II-α expression	Sunitinib
**14**	Moderately strong EGFR overexpression, RAS wild-type	Folinic acid, Fluorouracil and Irinotecan (FOLFIRI) and Cetuximab
**15**	Strong EGFR overexpression, RAS wild-type	Cisplatin and Gemcitabine
**16**	Strong EGFR overexpression, RAS wild-type, increased phosphorylation of ERK1/2 and Akt, moderately strong HER-2 overexpression (2+), no amplification, Ki-67 high	PIPAC (Pressurized Intra Peritoneal Aerosol Chemotherapy)
**17**	Strong EGFR overexpression, RAS wild-type, increased phosphorylation of ERK1/2, Ki-67 high, strong Topoisomerase-II-α overexpression	Vinorelbine and Cisplatin/Gemcitabine
**18**	Moderately strong EGFR overexpression, RAS wild-type, Increased phosphorylation of ERK1/2, MEK1/2 and Akt, moderately strong c-MET overexpression, Ki-67 high, low Topoisomerase-II-α expression	Fluorouracil, Leucovorin, Oxaliplatin and Docetaxel (FLOT)
**19**	Moderately strong EGFR overexpression, RAS wild-type, Ki-67 high, strong Topoisomerase-II-α overexpression	Regorafenib
**20**	Strong EGFR overexpression, RAS mutation status unknown, strong c-MET overexpression, no MET amplification, Ki-67 high, strong Topoisomerase-II-α overexpression	Folinic acid, Fluorouracil and Irinotecan (FOLFIRI)
**21**	Strong EGFR overexpression, KRAS mutation, moderately strong c-MET overexpression, no MET amplification, Ki-67 high, strong Topoisomerase-II-α overexpression	Folinic acid, Fluorouracil and Oxaliplatin (FOLFOX) and Bevacizumab
**22**	Strong EGFR overexpression, RAS wild-type, Ki-67 moderately high, low Topoisomerase-II-α expression	Palliative radiation
**23**	Strong EGFR overexpression, RAS wild-type, strong c-MET overexpression, no MET amplification	Nivolumab
**24**	Strong EGFR overexpression, RAS wild-type, activating EGFR mutation, Ki-67 high, strong Topoisomerase-II-α overexpression	Radiotherapy, six months later Erlotinib
**25**	Weak EGFR overexpression, RAS wild-type, moderately increased phosphorylation of MEK1/2	Adriamycin
**26**	Moderately strong EGFR overexpression, RAS wild-type, moderately strong c-MET overexpression, no MET amplification, increased phosphorylation of ERK1/2 and MEK1/2	Fluorouracil, Leucovorin, Oxaliplatin and Docetaxel (FLOT)
**27**	Moderately strong EGFR overexpression, KRAS mutation, strong c-MET overexpression, no MET amplification, moderately strong EML4-ALK overexpression, ALK FISH negative, increased phosphorylation of MEK1/2, Ki-67 moderately high, low Topoisomerase-II-α expression	Gemcitabine and nab-Paclitaxel
**28**	Moderately strong EGFR overexpression, RAS mutation status unknown, moderately strong c-MET overexpression, no MET amplification, moderately strong EML4-ALK overexpression, ALK FISH negative, Ki-67 high, strong Topoisomerase-II-α overexpression	Folinic acid, Fluorouracil and Irinotecan (FOLFIRI) and Bevacizumab

**Figure 5:**
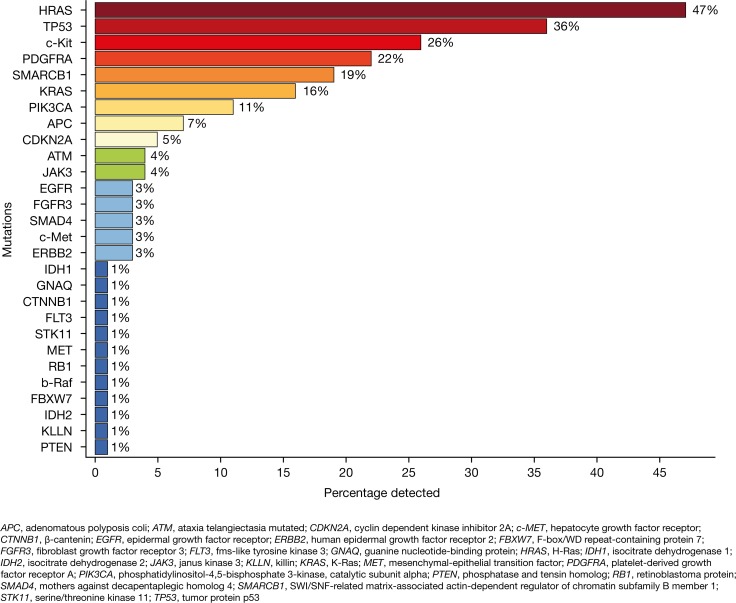
Frequency of identified mutations analyzed by next-generation sequencing (NGS) Analyses were performed for 73 patients dependent on the availability of tumor tissue with adequate tumor cell content.

Using NGS, the most commonly identified mutations were HRAS, TP53 and c-Kit which were detected in 47%, 36% and 26% of patients, respectively. Activating mutations indicating sensitivity to available targeted drugs or predictive mutations indicating the probability of response or resistance to drugs were found in the genes KRAS, PIK3CA, APC, EGFR, FLT3 and PTEN in a fraction of 16%, 11%, 7%, 3%, 1% and 1%, respectively (Figure 5). The corresponding COSMIC IDs of the identified mutations are summarized in Supplementary Table 1.

At six months, 49% of patients treated with targeted therapy based on molecular profiling had achieved disease control (CR, 14%; PR, 21%; SD, 14%; PD, 36%). In the other group, 21% of patients treated with non-targeted therapy achieved disease control (CR, 0%; PR, 0%; SD, 21%) while 64% showed PD. In both groups, two patients (14%) died within six months, irrespective of the initial treatment approach (Figure 3b).

**Figure 6:**
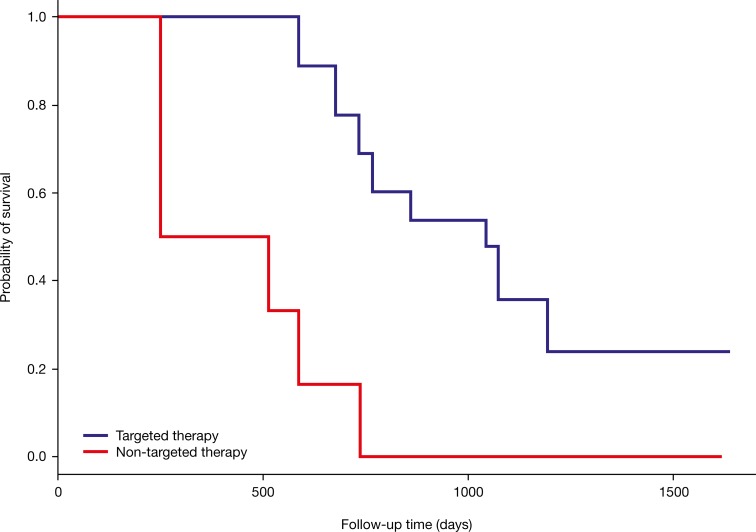
Kaplan-Meier survival curves of patients receiving targeted therapy based on molecular profiling (*N*=14) and those receiving non-targeted therapy independent of molecular profiling (*N*=14) The red line belongs to the 14 patients who were initially not treated according to a specific molecular target. The blue line belongs to the 14 patients who had an initial therapy matched to a molecular target.

Of the patients who were initially treated with targeted therapy based on molecular profiling, 9/14 (64%) survived ≥1 year from the point of time when the attending oncologist received their results, and 8/14 (57%) had an overall survival time of ≥1.5 years. While there was a trend towards improved overall survival with targeted therapy compared with non-targeted therapy, this did not reach statistical significance (*P*=0.23) (Figure 6). However, the median survival time in the group initially treated based on molecular profiling was approximately 3-fold greater compared with those treated with non-targeted therapy (2.9 years versus 1.1 years). Approximately 50% of patients initially treated with targeted therapy had at least two subsequent follow-up intervals without PD despite the initial poor prognosis. On the contrary, this was only true for 21% of patients receiving non-targeted therapy (Figure 3a and 3b).

For CUP, few guidelines are available indicating that molecular profiling for this tumor type may be particularly advantageous [12]. In total, 15 patients diagnosed with CUP who had developed liver and lymph node metastases and peritoneal carcinomatosis were included in this study. However, not every proteomic and genomic alteration could be matched to targeted therapies, and no amplification of human epidermal growth factor-2 (HER-2), epidermal growth factor receptor (EGFR), anaplastic lymphoma kinase (ALK) or c-MET was found by *in situ* hybridization (FISH or CISH). In addition, the majority of patients with CUP (8/15 [53%]) did not meet the criteria for molecular profiling as they died prior to the first follow-up or had no follow-up data (2/15 [13%]) and one of the probes was external. Overall, four patients with CUP (4/15 [27%]) were included in the follow-up analysis: one patient received targeted therapy based on molecular profiling for 3 months before the attending oncologist initiated chemotherapy, and three were treated with non-targeted therapy. Two patients were alive ≥9 months following molecular profiling independent of the initial therapy decision.

### Case reports

The following case reports are from the follow-up data of the attending physician/oncologist of patients receiving targeted therapy based on molecular profiling. Case numbers correspond to patient numbers in Table 1 and Figure 3a, and are selected examples of applied targeted treatment based on molecular profiling.

### Case #2

This patient was a 73-year-old male diagnosed with cholangiocarcinoma of the right hepatic lobe. Explorative laparotomy, a hemihepatectomy (right), a diaphragmatic resection, cholecystectomy and adrenalectomy were performed. The moderately differentiated cholangiocarcinoma with infiltration of the perihepatic soft tissue was treated with chemotherapy (gemcitabine/cisplatin) until CR, but liver metastases developed. Molecular profiling of the primary tumor and liver metastases was requested by the patient and three potential targets for targeted treatment were identified.

Wildtype for KRAS and a strong EGFR expression were detected in >90% of the tumor cells, thus addition of the EGFR monoclonal antibody cetuximab or panitumumab to a platinum-based chemotherapy was recommended. A combination of gemcitabine/cisplatin and cetuximab resulted in PR during the first 3 months of follow-up, but cetuximab was discontinued due to skin toxicity. During the following three months the disease progressed and the quantity and size of metastases increased despite changing therapy to capecitabine.

An identified KIT mutation led to the “off-label” use of imatinib, but despite promising data from the gastrointestinal stromal tumor (GIST), the patient did not achieve a positive clinical response. The patient then received the previously used chemotherapy consisting of gemcitabine and cisplatin with further changes to the chemotherapy including three cycles of FOLFIRI and a combination of gemcitabine and oxaliplatin. Finally, nivolumab was trialed, but showed no improvement. Additional identified alterations in the MET pathway as a moderately expressed c-MET receptor, an activation of the signaling protein Akt and an activating mutation of the PIK3CA gene were not considered for therapy decision by the attending oncologist.

### Case #9

This patient is a 51-year-old female diagnosed in July 1997 with breast cancer. A tumor of the right breast was surgically resected in July 1997, followed by radiotherapy. The tumor was histologically classified as a hormone receptor-negative adenocarcinoma (Grade 2) with unknown HER-2 expression. In July 2001, tumor recurrence developed in the right breast and chest wall, which was hormone receptor-negative but strongly HER-2 positive (3+). Breast removal was necessary, followed by adjuvant chemotherapy with epirubicin/cyclophosphamide (EC) from July 2001 to December 2001, adjuvant radiotherapy of the right chest wall from January 2002 to March 2002, and trastuzumab monotherapy from May 2002 to April 2004. Lymph node and bone metastases were found in January 2005, both hormone receptor-negative and HER-2 strongly positive (3+), leading to a therapy of radiation, trastuzumab and vinorelbine. This combination therapy resulted in SD for approximately two years.

In 2007, the cancer progressed with cutaneous metastasis with no change in hormone receptor or HER-2 expression. From 2007 to 2012, the patient received several single-agent chemotherapies, intermittently in combination with trastuzumab and bevacizumab. In 2013, the patient received trastuzumab emtansine (TDM-1) in the TH3RESA trial (NCT01419197) (TDM-1) [13], but no clinical response was observed. Afterwards the patient received a dual blockade of the HER-2 receptor dimerization with trastuzumab and pertuzumab, which resulted in a minor clinical response.

In June 2014, a dramatic progression of cutaneous metastasis was observed and the patient received opioid therapy. In July 2014, fresh biopsies of the metastasis were taken and our combined molecular profiling approach was applied. The following therapy options were available: vertical dual blockade of the HER-2 receptor due to strong HER-2 expression (3+); everolimus to inhibit the PI3K/Akt/mTOR pathway due to an activating mutation of the PIK3CA gene; topoisomerase inhibitor, as expression of topoisomerase was moderately high and the proliferation index of Ki-67 was moderately high to high; and EGFR antibodies, as EGFR receptor expression was strong and the RAS and EGFR mutation status was wildtype.

During the first six months following molecular profiling, the patient received a vertical dual blockade with trastuzumab, an inhibitor of MAPK and PI3K/Akt signaling and inducer of HER2 internalization [14, 15], and lapatinib which selectively targets and inhibits HER2 and EGFR [16–18]. This therapy resulted in CR with no side effects and discontinuation of opioid therapy. As the tumor progressed and cutaneous metastases developed, the therapy was changed to a combination of trastuzumab and capecitabine. Although the patient developed SD, the therapy was adapted over three months to a combination of trastuzumab and everolimus, analogous to the BOLERO-3 trial (NCT01007942) [19], and maintained for the following three months, then changed to trastuzumab and gemcitabine. Despite the patient having a very poor prognosis and receiving best supportive care, she has been successfully treated for >18 months since molecular profiling was performed on the fresh biopsies.

### Case #13

This patient is a 49-year-old male, diagnosed with metastasized, poorly differentiated neuroendocrine carcinoma of the pancreas in September 2013. The tumor progressed despite first-line therapy with carboplatin and etoposide, second-line therapy with capecitabine and temozolomide, third-line therapy with 5-FU and two cycles of streptozotocin, and fourth-line topotecan therapies. A clinical response was achieved after the therapy was changed to everolimus within the first three months of treatment.

Molecular profiling of a biopsy revealed increased PI3K/Akt/mTOR pathway activity and an activating mutation in the FLT3 gene. The patient continued to receive everolimus, then changed to sunitinib, a tyrosine kinase inhibitor (TKI), which resulted in PR.

## DISCUSSION

The aim of this study was to prove the advantage of a multi-omic approach compared with conventional strategies to select targeted therapies for patients with advanced stage IV cancer. This study was intended for patients with no further guideline-based therapy options, but was also performed if requested by the attending physician/oncologist in cases with very poor prognosis. Precision medicine must be built on understanding the complexity of the individual disease, therefore, our approach was based on tissue that was obtained under highly standardized conditions and includes tissue that has been snap frozen within seconds following biopsy to preserve the protein and phosphoprotein pattern [20]. This approach allowed us to interpret genetic alterations with reliable measured levels of targetable proteins and phosphoproteins that indicate activity of targetable pathways. Tumor tissue samples of metastases developed from different primary tumor types were collected from 83 patients. Molecular profiling consisting of (phospho-) proteomic and genomic methods was conducted to obtain a comprehensive overview of the molecular characteristics of the disease independent of the origin of the primary tumor. To obtain additional or novel insights into cancer on the molecular level for each patient, the same targets were analyzed whenever applicable including therapeutically relevant receptor proteins, signaling proteins of the MAPK and PI3K/Akt/mTOR pathways, and mutations in “hot spot” regions of 50 oncogenes and tumor suppressor genes. After applying the selected inclusion criteria, such as availability of follow-up data at ≥3 months and the ability to apply a comprehensive analysis, 28 patients were evaluable. The remaining analyzed patients were disclosed from the evaluation since they did not meet the determined criteria. For most patients at least one “druggable” target was found that offered further therapy options. Regardless of detected targets, some patients received for various reasons no targeted therapies (11/28 [39%]) or no additional alteration was detectable (3/28 [11%]). Therefore, 14 patients were treated according to identified alterations immediately after molecular profiling was completed. Targeted therapy was often administered in combination with chemotherapies. For all patients targeted treatment was decided by the attending physician/oncologist and if more than one potential target was identified and treatable, the sequence of treatment was their individual decision.

In ongoing trials, such as the Initiative for Molecular Profiling in Advanced Cancer Therapy (IMPACT) trial (NCT00851032) conducted by the MD Anderson Cancer Center Initiative which includes >1000 patients suffering from different types of advanced cancer, targeted treatment resulted in improved outcome [8], and this finding led to the extension of the study with the IMPACT 2 trial (NCT02152254). In both trials, molecular profiling is used to initiate targeted therapy and subsequently allow comparison with standard-of-care therapies to assess improvement in disease control [21, 22]. Although the molecular targets in the IMPACT and IMPACT 2 trials are limited to genetic alterations, the results affirm the benefits of precision oncology [8]. Although our study included less patients, a similar trend was observed as 64% (9/14) patients treated with targeted therapy according to their molecular profile showed overall survival times greater than 1 year. In contrast, the same observation could only be made for 29% (4/14) patients receiving non-targeted therapy independent of their molecular profile. Some patients included in our study had already started palliative treatment or best supportive care and had no expectations of recovery. For these patients, molecular profiling was a clear advantage because it offered further therapy options for them and prolonged survival time, and in single cases, CR was observed despite initial poor prognosis. Taking all follow-up cases into account, a significant superiority of our approach could not be proven but a positive trend was observed, which is already a remarkable outcome, in particular for individual patients (Figure 3a and Figure 6).

Further trials are recruiting patients to prove the impact of the integration of molecular targets into therapeutic decisions, such as the NCI-MATCH trial (NCT02465060). This trial will be conducted by the National Cancer Institute (NCI) and has an estimated enrollment of 6452 patients with advanced solid tumors who will be screened for genetic alterations to find a therapy matched to their molecular alteration. The intention of the NCI-MATCH trial is to evaluate the benefit of targeted therapies by assigning patients to different treatment subprotocols [23]. The SHIVA-trial, the first randomized controlled phase 2 trial showed that “off-label” use of molecularly targeted drugs outside their indication did not improve progression-free survival in comparison to treatment of physicians' choice [24]. These findings are contrary to the published data of other trials described above. As reasons were mentioned that many patients are unlikely to respond to monotherapies and single agents are ineffective in advanced cancer and some of the applied drugs were weak targeted drugs [25]. The WINTHER trial (NCT01856296; currently suspended), a multinational trial of the Worldwide Innovate Network (WIN) Consortium, will screen for DNA and RNA alterations of matched tumor and normal biopsies to identify molecular targets for targeted therapy selection of patients with metastatic cancer [26]. The WINTHER trial has an estimated enrollment of 200 patients, and at present is the only trial planning to integrate the transcriptomic data to select targeted therapies. These trials will provide novel insights into the molecular processes of cancer and will further support the benefit of targeted therapy based on molecular profiling.

In our study tissue samples of different tumor types were analyzed (Table 1). For all patients who received targeted therapy based on molecular profiling, it was an advantage to use three different methods to obtain a comprehensive overview of the properties of each tumor tissue sample. Identification of targets leading to therapeutic decisions in patients was based on immunohistochemistry, phosphoprotein profiling and NGS in 71%, 57% and 36% of cases, respectively (Figure 4). In summary, using three different methods increased the likelihood of finding a “druggable” target and allowed the identification of activating mutations as well as the expression status of receptor proteins in concordance with the phosphorylation/activation of signaling proteins of the analyzed MAPK and PI3K/Akt/mTOR pathways. The importance of analyzing the phosphorylation/activation of proteins of relevant signaling pathways in addition to the genetic changes and expression levels of receptor proteins has been shown to increase the quality of prediction of response to tyrosine kinase inhibitors (TKIs) e.g. in NSCLC [27]. Inhibitors of ERK, MEK, Akt and mTOR are potent anticancer drugs and are currently being investigated in clinical trials or are approved, such as the MEK inhibitor trametinib, indicated for unresectable or metastatic melanoma with BRAF V600E or V600K mutation [28] or the mTOR inhibitor everolimus, whose indications include advanced hormone receptor-positive, HER2-negative breast cancer, progressive neuroendocrine tumors of pancreatic origin and advanced renal cell carcinoma [29].

For CUP, few guidelines are available; therefore, using molecular profiling to define the metastases profile of this type of cancer is most appropriate [12]. Comprehensive genomic profiling of 200 patients with CUP in a study revealed clinically relevant genomic alterations in 85% of patients and for every case at least one identified alteration could be matched to targeted therapies or clinical trials [30]. In our study only four patients with CUP met the analytical criteria and could be evaluated; three patients were treated with non-targeted therapies as no targeted therapy could be identified and only one patient was treated with a targeted therapy. However, irrespective of therapy, two patients with CUP lived ≥9 months following initiation of treatment. Due to the small number of patients with CUP included in this study, the benefit of molecular profiling for patients with CUP is still to be determined.

In conclusion, this study offered valuable insights into the benefit of targeted therapy based on molecular profiling in patients with advanced stage IV cancer. But this pilot study also showed that it is of great importance to select patients before molecular testing is considered. 83 patients underwent molecular profiling, but most of them died shortly afterwards, which indicates that the interpretation of our inclusion criteria left much room for patients who were already in a too progressive condition of the disease to benefit from the findings. Therefore in the continuation of this approach a stringent structure should be built up to define the indication for molecular testing more precisely, e.g. through an outpatient therapy center to increase the chance for patients to obtain targeted agents for a sufficient period of time. Crucial for revealing drug targets for further standard therapies was a fresh biopsy, which led in single cases to CR. We are aware that our approach has to be adjusted to new upcoming methods and new insights regularly. One issue of our study was that only for 61% of the patients all intended methods were applicable. When the study was started in 2014, we used assays available at that time and kept it for the whole study to ensure comparability. We are currently applying and establishing new assays that require significantly less input material and therefore will be applicable for more samples. We suggest that this multi-omic approach will contribute to the identification of additional molecular targets already known to be significant for particular tumor types, as well as contribute to the identification of treatable alterations in tumor entities with unknown targets. An extension of this study with increased sample size will lead to affirming the benefits of molecular profiling and targeted therapy and enable significant improvements in overall survival to be observed in patients with advanced stage IV cancer.

## MATERIALS AND METHODS

### Patients

Patients were aged ≥18 years with advanced stage IV cancer of any tumor type. This pilot study was intended for patients without further guideline-based therapy options but was also performed if requested by the attending physician/oncologist in cases with very poor prognosis. All patients provided written informed consent and were in good general health, with an Eastern Cooperative Oncology Group (ECOG) performance status of 0 or 1 and a life expectancy ≥3 months. The study protocol was reviewed and approved by the competent ethics review committee of the Medical Association of Hamburg, Germany (Reference Number: PV5035).

Every three months, follow-up data were obtained relating to the patient's health status (improved, stabilized, worsened) and general condition (ECOG performance status and Karnofsky index; prior to and following therapy), as well as treatment course (types and changes to applied therapy, success of therapy and tumor progress) and whether a targeted therapy was being considered by the attending oncologist. In addition, use of radiotherapy (computed tomography [CT], microbeam radiation therapy [MRT]), reasons for any potential therapy pauses and side effects were also recorded. Disease progression was categorized as follows: complete remission (CR), no tumor detectable; partial remission (PR), tumor volume reduced by ≥50%; stable disease (SD) including minor response, change of tumor volume by <25% and tumor volume reduced by 25-49%, respectively; progressive disease (PD), tumor volume increased by ≥25%.

### Tissue collection and processing

Tissue collection of all 83 cases was performed under highly standardized conditions used by Indivumed, GmbH, as described previously [10, 31]. Tissue collection of the evaluable cases was mainly carried out at hospitals in Hamburg, Germany, namely Agaplesion Diakonieklinikum; Albertinen-Krankenhaus, Asklepios Klinik (Altona, Barmbek and Harburg); Israelitisches Krankenhaus; Krankenhaus Jerusalem, Marienkrankenhaus and Universitätsklinikum Hamburg-Eppendorf. Further hospitals included Klinikum Vest in Marl and Recklinghausen and Prosper-Hospital in Recklinghausen. Tissues were processed using an automated system (Microm Tissue Processor STP420 D, Thermo Scientific, Dreieich, Germany) and embedded in paraffin (Paraplast^®^, Carl Roth GmbH, Karlsruhe, Germany). To ensure quality control and the feasibility of the analytical tests, tumor cell content was ≥10% and ≥60% for formalin-fixed paraffin-embedded (FFPE) and fresh frozen (FF) tissues, respectively. Formalin fixation time was 4-24 hours, depending on sample size; however, for phosphoprotein measurements, the maximum tissue freezing time was 10 minutes, due to rapid changes in phosphoprotein activity. Tumor cell enrichment through macro-or microdissection with laser capture was performed where possible. Main tumor types of interest included cancer of unknown primary (CUP), colorectal cancer, esophageal carcinoma/gastric cancer, gall bladder-/cholangiocarcinoma, pancreatic cancer, and triple-negative breast cancer (TNBC).

### Immunohistochemistry

Tumor samples (3 μM sections of FFPE) were prepared and mounted on Superfrost™ Ultra Plus^®^ Adhesion Slides (Thermo Fisher Scientific, Waltham, MA, USA) and immunohistochemically stained using the automated Ventana BenchMark ULTRA Slide Staining System (Ventana Medical Systems, Inc., Tucson, AZ, USA). Assays were conducted using primary antibodies against: echinoderm microtubule-associated protein-like 4– anaplastic lymphoma kinase (EML4–ALK [ALK (D5F3) XP]); epidermal growth factor receptor (EGFR [EGFR CONFIRM anti-EGFR (3C6)]); human epidermal growth factor receptor 2 (HER-2 [PATHWAY anti-HER-2/neu (4B5)]); insulin-like growth factor 1 receptor (IGF-1R [CONFIRM anti-IGF-1R (G11)]); Ki-67 (CONFIRM anti-Ki-67 (30-9)]); programmed death-ligand 1 (PD-L1 [SP263]); topoisomerase-II-α (TOP2A [CONFIRM anti-topoisomerase-II-α (JS5B4)]); tyrosine-protein kinase Met (c-MET [CONFIRM anti-Total c-MET (SP44)]); and vascular endothelial growth factor receptor 2 (VEGFR-2 [VEGF Receptor 2 (55B11)]). All primary antibodies were rabbit monoclonal antibodies, except anti-EGFR which was a mouse monoclonal antibody, and were purchased from Ventana Medical Systems Inc. (Tucson, AZ, USA), except for EML4–ALK and VEGFR-2 (purchased from Cell Signaling Technology Inc., Danvers, MA, USA). HER-2 chromogenic *in situ* hybridization (CISH) (INFORM HER-2 Dual ISH DNA Probe Cocktail Assay), EGFR-fluorescence *in situ* hybridization (FISH), c-MET FISH and EML4-ALK FISH (from an external provider), were used if required. Following staining, the slides underwent an ascending alcohol series and were covered with Pertex (Medite GmbH, Burgdorf, Germany). An indirect biotin-free system was used for the detection of rabbit primary antibodies (ultraView Universal DAB and optiView DAB IHC Detection Kit). For the mouse primary antibody EGFR (3C6) the iView DAB Detection kit, an indirect biotin streptavidin system was used. Evaluation and interpretation of results were performed by a pathologist.

### Next-generation sequencing (NGS)

For DNA sequencing, the Ion AmpliSeq™ Cancer Hotspot Panel v2 (Life Technologies, Carlsbad, CA, USA) was applied covering “hotspot” regions of 50 oncogenes and tumor suppressor genes including: ABL1, AKT1, ALK, APC, ATM, BRAF, CDH1, CDKN2A, CSF1R, CTNNB1, EGFR, ERBB2, ERBB4, EZH2, FBXW7, FGFR1, FGFR2, FGFR3, FLT3, GNA11, GNAS, GNAQ, HNF1A, HRAS, IDH1, JAK2, JAK3, IDH2, KDR, KIT, KRAS, MET, MLH1, MPL, NOTCH1, NPM1, NRAS, PDGFRA, PIK3CA, PTEN, PTPN11, RB1, RET, SMAD4, SMARCB1, SMO, SRC, STK11, TP53, VHL. FF tissue was preferred to FFPE tissue. NGS analyses were conducted by an external provider (Labor Dr. Fenner und Kollegen, MVZ für Labormedizin und Humangenetik GmbH, Molekulargenetik, Hamburg, Germany).

### Phosphoprotein profiling (NanoPro™ 1000)

Phosphorylated protein of extracellular signal-regulated protein kinases 1/2 (ERK1/2), mitogen-activated protein kinase kinase (MEK1/2) of the MAPK pathway, and Akt of the PI3K/Akt/mTOR pathway were analyzed using NanoPro™ 1000 Technology (Protein Simple, Santa Clara, California, USA). To detect total protein and phosphorylated isoforms, pan antibodies were applied against: ERK (ready-to-use ERK; Protein Simple, Santa Clara, CA, USA), MEK1/2 and Akt (both Cell Signaling Technology Inc., Danvers, MA, USA). Phosphoprotein profiling was conducted by Indivumed GmbH, Hamburg, Germany.

### Survival analysis

The R package *survival* was applied for the survival analysis. Kaplan-Meier survival curves were created from a model formula in which the response variable was based on the starting and ending times of the follow-up intervals, as well as the event of dying [32, 33]. To determine significant differences of survival times between patient subgroups, a Cox proportional hazards regression model was fitted [34]. To test the null hypothesis that the hazard ratio equals one, the Wald test was used [35].

## SUPPLEMENTARY MATERIALS TABLE





## Abbreviations

NGS: next-generation sequencing
MAPK: mitogen-activated protein kinase
PI3K: phosphoinositide 3-kinase
Akt: protein kinase B
mTOR: molecular target of rapamycin
NSCLC: non-small cell lung carcinoma
ECOG: Eastern Cooperative Oncology Group
CT: computed tomography
MRT: microbeam radiation therapy
CR: complete remission
PR: partial remission
SD: stable disease
PD: progressive disease
FFPE: formalin-fixed paraffin-embedded
FF: fresh frozen
CUP: cancer of unknown primary
TNBC: triple-negative breast cancer
RCC: renal cell carcinoma
IHC: immunohistochemistry
FISH: *Fluorescence in situ hybridization*
CISH: *Chromogenic in situ hybridization*
EML4-ALK: echinoderm microtubule-associated protein-like 4–anaplastic lymphoma kinase
EGFR: epidermal growth factor receptor
HER-2: human epidermal growth factor receptor 2
IGF-1R: insulin-like growth factor 1 receptor
PD-L1: programmed death-ligand 1
TOP2A: topoisomerase-II-α
c-MET: tyrosine-protein kinase Met
VEGFR-2: vascular endothelial growth factor receptor 2
FISH: fluorescence *in situ* hybridization
ERK1/2: extracellular signal-regulated protein kinases 1/2
MEK1/2: mitogen-activated protein kinase kinase
TKI: tyrosine kinase inhibitors
TDM-1: trastuzumab emtansine
EC: epirubicin/cyclophosphamide
FOLFIRI: Folinic acid, Fluorouracil and Irinotecan
FU: Fluorouracil
FOLFOX: Folinic acid, Fluorouracil and Oxaliplatin)
FLOT: Fluorouracil, Leucovorin, Oxaliplatin and Docetaxel
PIPAC: Pressurized Intra Peritoneal Aerosol Chemotherapy
