# Supplementary material for: Multi-omic based molecular profiling of advanced cancer identifies treatable targets and improves survival in individual patients
Source: Oncotarget. 2018 Oct 5;9(78):34794–809. doi: 10.18632/oncotarget.26198 (PMC6205171; doi:10.18632/oncotarget.26198)
Supplement: Supplementary file 1 [file oncotarget-09-34794-s001.pdf]

Multi-omic based molecular profiling of advanced cancer identifies treatable targets and improves survival in individual patients

SUPPLEMENTARY MATERIALS

Supplementary Table 1: Overview of all unique detected mutations (COSMIC IDs) and the amount of patients who harbored the identified mutation at least once

See Supplementary File 1
