# Supplementary material for: Multi-omic based molecular profiling of advanced cancer identifies treatable targets and improves survival in individual patients
Source: Oncotarget. 2018 Oct 5;9(78):34794–809. doi: 10.18632/oncotarget.26198 (PMC6205171; doi:10.18632/oncotarget.26198)
Supplement: Supplementary file 2 [file oncotarget-09-34794-s002.docx]

**Supplementary Table 1:** Overview of all unique detected mutations (COSMIC IDs) and the amount of patients who harbored the identified mutation at least once.

| \| Gene \| COSMIC ID \| Amount \| \| --- \| --- \| --- \| \| APC \| COSM13125 \| 2 \| \| APC \| COSM13127 \| 2 \| \| APC \| COSM19099 \| 1 \| \| ATM \| COSM22507 \| 3 \| \| b-Raf \| COSM476 \| 1 \| \| CDKN2A \| COSM13504 \| 1 \| \| CDKN2A \| COSM12473 \| 1 \| \| CDKN2A \| COSM12475 \| 1 \| \| CDKN2A \| COSM12491 \| 1 \| \| c-Kit \| COSM28026 \| 12 \| \| c-Kit \| COSM21983 \| 7 \| \| c-Kit \| COSM1155 \| 1 \| \| c-Met \| COSM707 \| 2 \| \| c-Met \| COSM1666978 \| 1 \| \| CTNNB1 \| COSM5684 \| 1 \| \| EGFR \| COSM6223 \| 1 \| \| EGFR \| COSM6224 \| 1 \| \| ERBB2 \| COSM14060 \| 1 \| \| ERBB2 \| COSM20959 \| 1 \| \| FBXW7 \| COSM22973 \| 1 \| \| FGFR3 \| COSM24842 \| 1 \| \| FGFR3 \| COSM724 \| 1 \| \| FLT3 \| COSM19692 \| 1 \| \| GNAQ \| COSM28757 \| 1 \| \| HRAS \| COSM249860 \| 34 \| \| IDH1 \| COSM1741220 \| 1 \| \| IDH2 \| COSM33733 \| 1 \| \| JAK3 \| COSM34213 \| 3 \| \| KLLN \| no COSMIC ID assigned \| 1 \| \| KRAS \| COSM521 \| 4 \| \| KRAS \| COSM518 \| 2 \| \| KRAS \| COSM532 \| 2 \| \| KRAS \| COSM555 \| 1 \| \| KRAS \| COSM19404 \| 1 \| \| KRAS \| COSM520 \| 1 \| \| KRAS \| COSM516 \| 1 \| \| MET \| no COSMIC ID assigned \| 1 \| \| PDGFRA \| COSM22413 \| 16 \| \| PIK3CA \| COSM760 \| 3 \| \| PIK3CA \| COSM21451 \| 2 \| | \| Gene \| COSMIC ID \| Amount \| \| --- \| --- \| --- \| \| PIK3CA \| COSM249908 \| 1 \| \| PIK3CA \| COSM776 \| 1 \| \| PIK3CA \| COSM775 \| 1 \| \| PIK3CA \| COSM27502 \| 1 \| \| PTEN \| no COSMIC ID assigned \| 1 \| \| RB1 \| COSM895 \| 1 \| \| SMAD4 \| COSM14122 \| 2 \| \| SMARCB1 \| COSM1090 \| 14 \| \| STK11 \| COSM21360 \| 1 \| \| TP53 \| COSM10662 \| 2 \| \| TP53 \| COSM10704 \| 2 \| \| TP53 \| COSM44093 \| 1 \| \| TP53 \| COSM44794 \| 1 \| \| TP53 \| COSM121035 \| 1 \| \| TP53 \| COSM10722 \| 1 \| \| TP53 \| COSM179822 \| 1 \| \| TP53 \| COSM10659 \| 1 \| \| TP53 \| COSM10813 \| 1 \| \| TP53 \| COSM99720 \| 1 \| \| TP53 \| COSM10810 \| 1 \| \| TP53 \| COSM44601 \| 1 \| \| TP53 \| COSM44321 \| 1 \| \| TP53 \| COSM45896 \| 1 \| \| TP53 \| COSM10887 \| 1 \| \| TP53 \| COSM11355 \| 1 \| \| TP53 \| COSM44657 \| 1 \| \| TP53 \| COSM99618 \| 1 \| \| TP53 \| COSM10648 \| 1 \| \| TP53 \| COSM11582 \| 1 \| \| TP53 \| COSM43545 \| 1 \| \| TP53 \| COSM45230 \| 1 \| \| TP53 \| COSM99668 \| 1 \| \| TP53 \| COSM43570 \| 1 \| \| TP53 \| COSM10992 \| 1 \| \| TP53 \| COSM43776 \| 1 \| \|  \|  \|  \| \|  \|  \|  \| \|  \|  \|  \| \|  \|  \|  \| \|  \|  \|  \| \|  \|  \|  \| |
| --- | --- | --- | --- | --- | --- | --- | --- | --- | --- | --- | --- | --- | --- | --- | --- | --- | --- | --- | --- | --- | --- | --- | --- | --- | --- | --- | --- | --- | --- | --- | --- | --- | --- | --- | --- | --- | --- | --- | --- | --- | --- | --- | --- | --- | --- | --- | --- | --- | --- | --- | --- | --- | --- | --- | --- | --- | --- | --- | --- | --- | --- | --- | --- | --- | --- | --- | --- | --- | --- | --- | --- | --- | --- | --- | --- | --- | --- | --- | --- | --- | --- | --- | --- | --- | --- | --- | --- | --- | --- | --- | --- | --- | --- | --- | --- | --- | --- | --- | --- | --- | --- | --- | --- | --- | --- | --- | --- | --- | --- | --- | --- | --- | --- | --- | --- | --- | --- | --- | --- | --- | --- | --- | --- | --- | --- | --- | --- | --- | --- | --- | --- | --- | --- | --- | --- | --- | --- | --- | --- | --- | --- | --- | --- | --- | --- | --- | --- | --- | --- | --- | --- | --- | --- | --- | --- | --- | --- | --- | --- | --- | --- | --- | --- | --- | --- | --- | --- | --- | --- | --- | --- | --- | --- | --- | --- | --- | --- | --- | --- | --- | --- | --- | --- | --- | --- | --- | --- | --- | --- | --- | --- | --- | --- | --- | --- | --- | --- | --- | --- | --- | --- | --- | --- | --- | --- | --- | --- | --- | --- | --- | --- | --- | --- | --- | --- | --- | --- | --- | --- | --- | --- | --- | --- | --- | --- | --- | --- | --- | --- | --- | --- | --- | --- | --- | --- | --- | --- | --- | --- | --- | --- | --- | --- | --- | --- | --- | --- | --- | --- | --- |
